# In vivo cross-sectional imaging of the phonating larynx using long-range Doppler optical coherence tomography

**DOI:** 10.1038/srep22792

**Published:** 2016-03-10

**Authors:** Carolyn A. Coughlan, Li-dek Chou, Joseph C. Jing, Jason J. Chen, Swathi Rangarajan, Theodore H. Chang, Giriraj K. Sharma, Kyoungrai Cho, Donghoon Lee, Julie A. Goddard, Zhongping Chen, Brian J. F. Wong

**Affiliations:** 1 Department of Otolaryngology-Head and Neck Surgery, University of California, Irvine, School of Medicine University of California, Irvine, CA, United States; 2 Beckman Laser Institute & Medical Clinic, University of California, Irvine, CA, United States; 3 OCT Medical Imaging Inc., University of California, Irvine, CA, United States; 4 Department of Biomedical Engineering, University of California, Irvine, CA, United States; 5 Department of Otorhinolaryngology-Head & Neck Surgery, Sanggye Paik Hospital, Inje University, Korea

## Abstract

Diagnosis and treatment of vocal fold lesions has been a long-evolving science for the otolaryngologist. Contemporary practice requires biopsy of a glottal lesion in the operating room under general anesthesia for diagnosis. Current in-office technology is limited to visualizing the surface of the vocal folds with fiber-optic or rigid endoscopy and using stroboscopic or high-speed video to infer information about submucosal processes. Previous efforts using optical coherence tomography (OCT) have been limited by small working distances and imaging ranges. Here we report the first full field, high-speed, and long-range OCT images of awake patients’ vocal folds as well as cross-sectional video and Doppler analysis of their vocal fold motions during phonation. These vertical-cavity surface-emitting laser source (VCSEL) OCT images offer depth resolved, high-resolution, high-speed, and panoramic images of both the true and false vocal folds. This technology has the potential to revolutionize in-office imaging of the larynx.

The vocal fold is a complex viscoelastic structure with multiple distinct tissue layers, which differ in both their optical and mechanical properties. *In vivo* imaging has largely been relegated to evaluation of surface morphology using classic mirror examination or conventional fiber-optic and rigid endoscopy. Functional imaging of vocal fold mechanics can be accomplished using stroboscopic methods or high-speed digital imaging, and both facilitate analysis of the vocal fold mucosal wave propagation during phonation. Regardless, all clinically used technology to date images only the surface, and hence can only provide clues with respect to the underlying sub-epithelial tissue structure.

Determining the cross-sectional microanatomy *in vivo*, particularly during phonation, will lead to a better understanding of vocal fold mechanics and provides a means to more reliably diagnose vocal fold pathology without biopsy. The vocal fold is a functional layered structure with spatially varying mechanical properties that vibrate with airflow, leading to phonation, a process that produces multiple harmonics and generates normal speech. There is a wide spectrum of disease that can involve the sub-epithelial region of the vocal folds. This includes benign (cysts, polyps), premalignant (dysplasia), and malignant lesions. A biopsy under general anesthesia is required to histologically differentiate these lesions. Unfortunately, biopsy of the vocal folds can risk creating permanent damage to the vocal fold and voice quality^1^. *In vivo* high-resolution imaging of the vocal fold would be of tremendous value for both clinicians and scientists.

Optical coherence tomography (OCT) is an imaging modality that acquires high-resolution cross-sectional images of living tissues, and has become the standard for retinal imaging in ophthalmology. It also has emerging applications in cardiology where it has been used to image plaque in the coronary vasculature. In the head, neck, and upper airway, OCT as an investigational technology has focused on imaging early cancers and dysplastic changes along the mucosa. Although several basic studies and clinical investigations have focused on examining human vocal fold structure, they have necessarily been performed primarily under general anesthesia^2^,^3^,^4^,^5^,^6^,^7^,^8^,^9^,^10^,^11^,^12^,^13^,^14^,^15^,^16^. As a high-resolution and non-contact imaging modality, OCT is an ideal technology to image the layered structure of the vocal folds *in vivo*.

Applications and technology development for laryngeal OCT imaging has been fairly limited because it typically must be accomplished in contact or near-contact mode, and in general this requires sedation and surgical endoscopy. Moving this technology from the operating room to the office has been an objective for several research groups for well over a decade^10^,^17^,^18^,^19^,^20^,^21^,^22^,^23^. Office-based imaging has remained a challenge as devices suffered from inadequate fields of view, limited imaging range, and inability to compensate for the subject’s head and examiner’s hand motion. Clinical studies have not yet produced meaningful cross-sectional image data, and hence device development and clinical adoption has not occurred. While vocal fold structure and motion has been recorded previously, true panoramic images of the vocal fold in cross-section while at rest and during phonation have not been reported^21^,^24^.

Here we report the first full frame, *in vivo*, cross-sectional imaging of the vocal folds during phonation using an OCT system incorporating a vertical-cavity surface-emitting laser source (VCSEL). The VCSEL source extends the imaging range and solves many of the problems that have limited laryngeal OCT development. We demonstrated in healthy normal volunteers: 1) the real-time, cross sectional structure of both the true and false vocal folds, 2) the native vibration of the vocal folds, and 3) the Doppler shift induced by the vocal fold vibration *in vivo*.

## Methods

### Clinical Parameters

This study was performed with the approval of the Institutional Review Board at the University of California, Irvine, and all methods were carried out in accordance with the approved guidelines. Healthy volunteers were recruited for participation, and written informed consents were obtained from all subjects. Potential volunteers were first examined with the intent of identifying favorable anatomy. Individuals with a very low palatal arch, posteriorly displaced base of tongue, or vivid gag reflex were excluded from participation. Ideal anatomy for this study focused on individuals who had a Mallampati class one or two airway. Volunteers were not excluded on the basis of race, age, or gender. No volunteer had a history of a voice, speech, or swallowing complaint.

**Figure 1:**
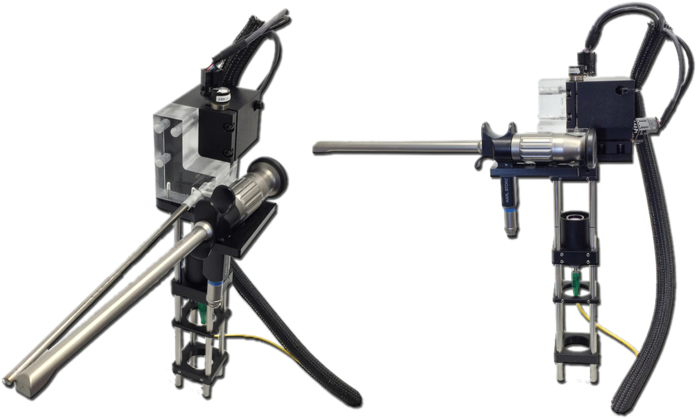
OCT hand piece. The OCT device mounted concurrently with a rigid laryngoscope.

Approximately 5 minutes prior to imaging, 4% lidocaine was delivered onto the surface the oropharynx and palate using an atomizer. Imaging was performed with the use of the tandem OCT-Video (Fig. 1).

### System Setup

**Figure 2:**
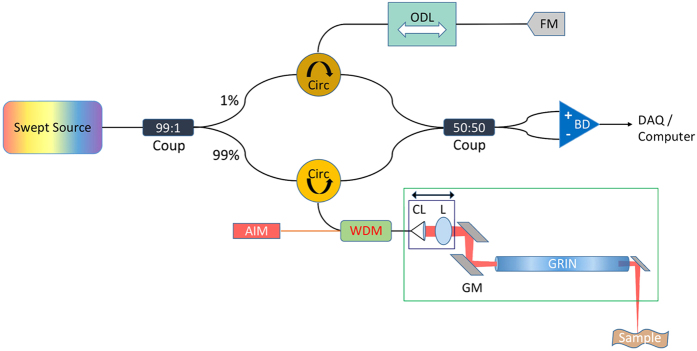
OCT System Schematics. Circ = fiber-optic circulator; ODL = optical delay line; FM = fiber mirror; BD = balanced detector; AIM = aiming laser; WDM = wavelength division multiplexer; CL = collimating Lens; L = imaging lens; GM = Galvo mirrors; GRIN = gradient index lens rod.

The swept source OCT system (Fig. 2) consisted of a 1310 nm center wavelength 200 kHz VCSEL swept source (SL1310V1-20048, Thorlabs, Inc., MA, USA), and the output light from the swept source was split by a 99:1 ratio fiber-optic coupler into sample and reference arms, respectively. The system was designed to ensure it had a symmetrical structure with identical circulators on both arms. In the reference arm, a faraday mirror was used to reflect the reference beam, while an optical delay line (ODL) was used to adjust and compensate for changes in sample arm path length. In the sample arm, a wavelength division multiplexer (WDM) was used to combine a 635 nm aiming laser beam with the 1310 nm sample beam from port 2 of the circulator before the laryngeal scanner setup. This allowed visualization of the scanning region during OCT imaging. The laryngeal setup (Fig. 1) consisted of a fiber collimator and an achromatic doublet focusing lens, a 2-axis galvo mirrors, a gradient index (GRIN) lens rod, and a 45-degree reflector. The GRIN lens rod and the reflector were housed inside a stainless steel tube with 5.2 mm outer diameter. The aforementioned combined sample beam and aiming laser beam were sent through the WDM to the fiber collimator. This collimated output beam was subsequently focused by the achromatic doublet (f = 125 mm), and directed towards the proximal end of the GRIN lens rod by the 2-axis galvo mirrors.

The focusing sample beam was then scanned by the 2-axis galvo and translated to the distal end of the 1 pitch GRIN lens rod, where the beam was reflected 90 degrees downward by the 45-degree reflector. The fiber collimator and achromatic doublet were mounted in the same subassembly and could be adjusted to tune the focal plane position of the sample beam. The focal plane could range from 30 mm to 100 mm to accommodate the anatomical differences in mid-oropharynx to vocal fold distance between test subjects. This plane was preset to approximately 60 mm from the tip of the laryngeal setup, where the beam spot size was 74.5 μm and corresponding depth of focus was 6.4 mm as measured by a beam profiler (DataRay Inc., Bella Vista, California). This configuration allowed more than half of the imaging range (12 mm) to be in focus. Using a single reflector as sample, the axial resolution of the system was measured to be approximately 12.5 μm in air, or 9.3 μm in tissue. In contrast, lateral resolution is diffraction limited and is a function of the design of the focusing optics. With the present device, lateral resolution at 10 cm is 100 μm. Displaying data with anatomically correct aspect ratios subjectively reduces the image quality as pixels are “stretched” laterally. This does create a perception of lower resolution to the naked eye. Here the real-time OCT images are presented with the aspect ratio corrected.

The 2-axis galvo mirrors were positioned after the focusing lens such that the lateral scanning distance varied with the working distance, thereby creating a fan-shaped cross-sectional scanning area. For a 60 mm working distance from the tip of laryngeal setup, the scanning range varied from 7.8 mm at the top to 9.2 mm at the bottom of an OCT image. For this study, a fixed lateral frame size of 1000 a-lines was selected to achieve a frame rate of 200 Hz.

### Image acquisition

**Figure 3:**
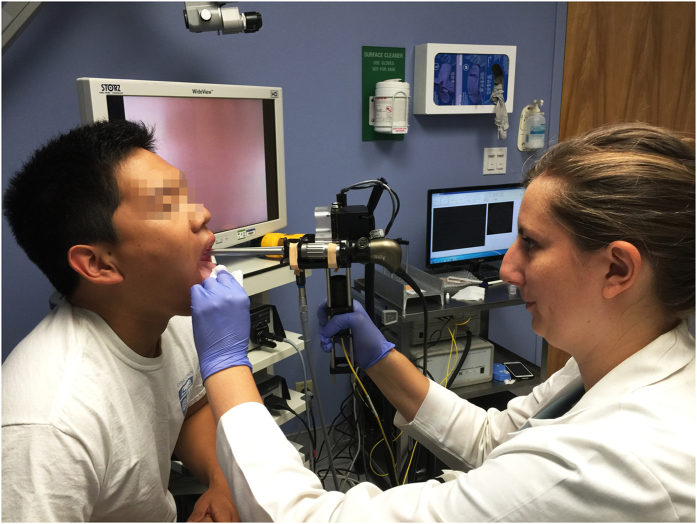
Imaging procedure. As the operator is instructing the participant, she is able to drive the laryngoscope using the endoscopy video. At the same time, OCT data is collected. By visualizing the red light bar, the operator is able to aim the OCT laser to any area of concern.

Simultaneous OCT and conventional video images were captured using a tandem device (Figs 1 and 3) modeled after previous units we had designed. The conventional endoscope used was a 90° rigid laryngoscope (Storz, Tuttlingen, Germany, #8707 DA). This device has a 4x objective with a diameter of 10 mm and length of 15 cm. This endoscope was mounted into the same bracket as the OCT optics so that the two devices could be used simultaneously. The operator was able to see the region of the vocal fold scanned due to a red aiming beam from a diode laser, which was scanned along with the beam from the OCT system. The aiming beam identified the region of the vocal fold being imaged and also secondarily functioned as a target for both conventional endoscope and the OCT beam to focus.

Using a digital camera (iPhone 6, Apple, California), audio and video data was recorded during the imaging session while the subject was phonating. The audio information was extracted and fast Fourier Transform (FFT) algorithm was used to analyze the fundamental frequency of the data.

The OCT images were processed with a 10.75:1 width-to-height ratio to roughly account for the difference in axial and lateral resolutions. The lateral resolution is approximately 100 μm per pixel in tissue. The threshold was set at 50% to eliminate the speckle noise in the background. Images are presented using a red-to-yellow gradient map. To increase the contrast, the images were adjusted so that 1% of data is saturated at low and high intensities.

## Results

### Vocal fold structure

**Figure 4:**
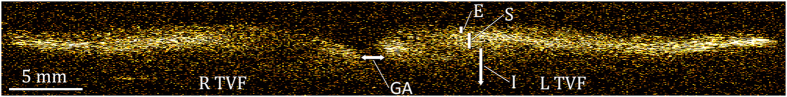
OCT B-frame of the subject phonating at approximately 850 Hz. R TVF = right true vocal fold; L TVF = left true vocal fold; GA = glottic aperture; E = epithelium; S = superficial lamina propria; I = intermediate lamina propria.

**Figure 5:**
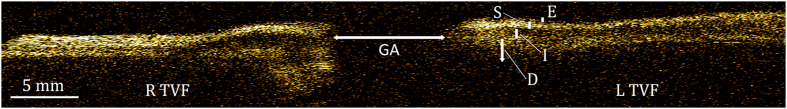
OCT B-frame of the subject phonating at approximately 250 Hz. The asymmetry of the left and right true vocal folds were due to the angled device during imaging. D = deep lamina propria.

Figure 4 is a single frame in the video sequence of a subject phonating at approximately 250 Hz, and Fig. 5 is a single frame in another video sequence of a subject phonating at approximately 850 Hz. As shown in Figs 4 and 5, in the cross-sectional images (B-scans), clear demarcations are visualized between the tissue layers. The most superficial layer (80–100 μm thickness), is the epithelium, and is consistent with previous OCT vocal folds measurements^16^. Beneath the epithelium is the superficial lamina propria (SLP), denoted by a band of increased pixel intensity. Between the epithelium and SLP is the basement membrane (BM). The BM is not resolved with OCT as it is a fibrous layer two cells thick. In the SLP, there is an increase in optical scattering that may be due to the increased collagen density in the SLP layer as well as the presence of a microvasculature. It was found to be approximately 300 μm in thickness. The intermediate lamina propria (ILP) is known to be immediately deep to the SLP and is histologically characterized by its less densely organized structure. This reduces the back-scattered signal, which correlates to its decreased pixel intensity. This layer may extend at least 650 μm, though signal intensity does drop significantly with depth. Our thickness measurements concur with the quantitative lamina propria analysis done by Prades *et al*. as well as studies on collagen in human vocal folds by others^25^,^26^,^27^.

A video (Supplementary Video 1) of the subject in Fig. 4 illustrates vocal fold vibration of a subject phonating at approximately 850 Hz. The epithelium, SLP, and ILP are clearly visualized as is the motion of vocal fold itself. The video is slowed as the true frame rate is 200 Hz. Of note, the Nyquist frequency is 1700 Hz, hence at this speech frequency aliasing effects occur with our system and do not allow illustration of the vibratory cycle. Supplementary Video 2 is a video clip of the subject illustrated in Fig. 5 phonating at 250 Hz. In this case, the superficial most portion of the deep lamina propria (DLP) can be observed. The complete DLP layer is not imaged. Since the frame rate is still below the Nyquist limit, the left and right sides of the image represent data acquired at different points in the vibratory cycle, which is seen as a lack of left to right symmetry. However, we are able to see the “mucosal wave” propagate in these images^28^.

### Vibration of the vocal folds

**Figure 6:**
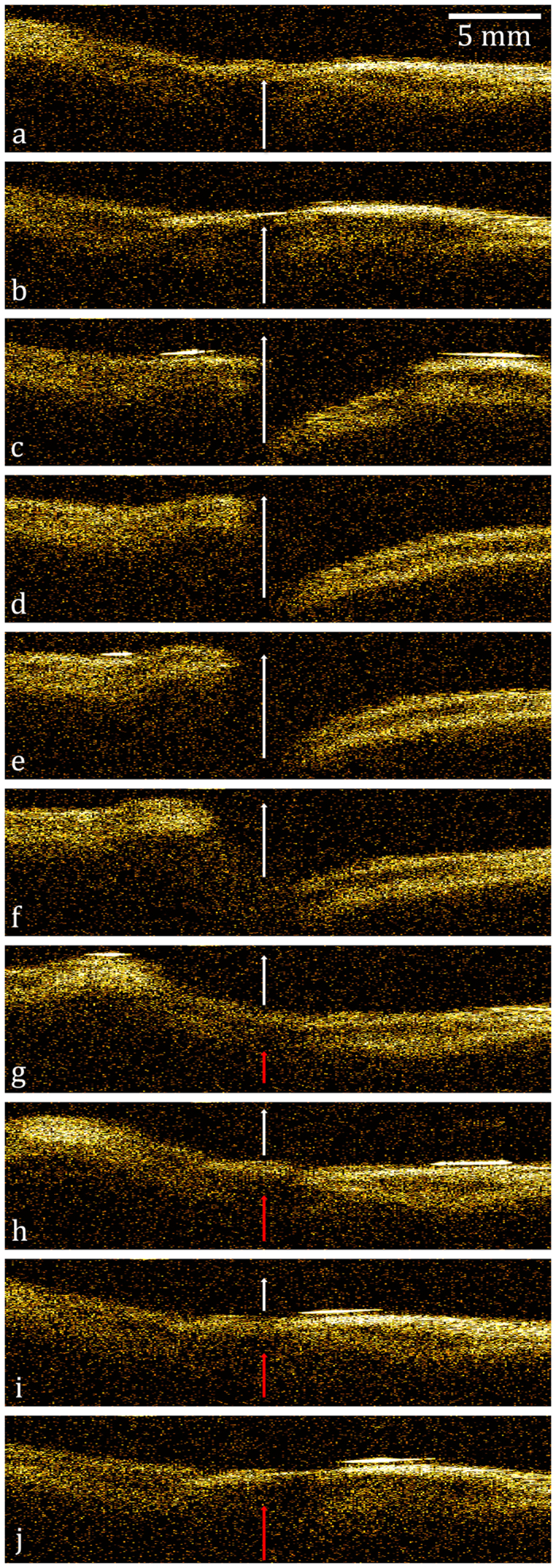
B-frame series of the subject phonating at roughly 250 Hz. The interval between each image is 5 ms. The arrows represent the direction of the air pulse.

A montage of B-scan images of the subject phonating at approximately 250 Hz is shown in Fig. 6. The phasic movement of the true vocal folds can be clearly observed. Figure 6a,b show the effect of positive air pressure built during vocal fold closure. Figure 6c–f illustrate vocal fold free margin movement during the release of the air pulse due to the pressure build up. As shown in Fig. 6g,j, the lower pressure at the end of the air pulse results in closure of the vocal folds and allows another air pressure column to build up, concluding a vibratory cycle. These series of images, spanning 50 ms, are supported by the real-time endoscopic video of the vocal folds (Supplementary Video 2) and is consistent with known physiology of phonation^24^,^29^. The B-scan images and the corresponding simultaneous conventional endoscopic video are presented side-by-side (Supplementary Video 3). In Supplementary Video 3, the OCT images are laterally compressed for easier visualization with endoscopy images. The red line visualized perpendicular to the vocal folds at the midpoint is a diode laser aiming beam used to aid the examiner in aiming the OCT beam.

### The Doppler shift induced by the vocal fold vibration *in vivo*

**Figure 7:**
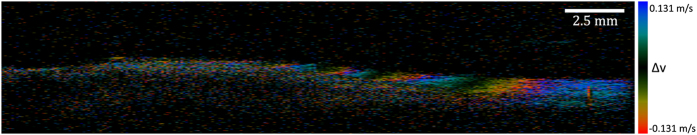
Doppler image of a B-frame. The subject was phonating at about 250 Hz. The red pixels represent movement towards the source and the blue regions show movement away from the source. The velocity distribution pattern can be easily observed on the downslope of the vocal folds; this result agree with the study done by Liu *et al*.^24^.

**Figure 8:**
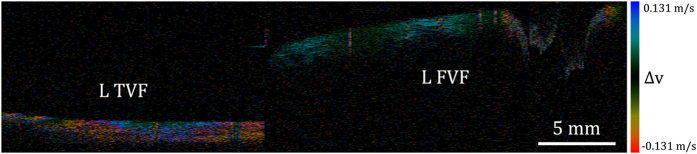
Doppler image of a B-frame. The subject was phonating at about 250 Hz. The image shows both left true and false vocal folds. The velocity distribution patterns are seen in both folds. However, the magnitude in the false vocal fold is much less than the true vocal fold. L TVF = left true vocal fold; L FVF = left false vocal fold.

The Doppler shift provides information on local tissue motion in and out of the direction of light propagation, and is calculated by computing the phase shift between adjacent A-lines within the same image (Fig. 7)^24^. The velocity distribution of the vocal folds is presented in colors (Fig. 7). By the Doppler convention, the red pixels represent regions moving towards the laser source while the blue denote areas moving away from the source. Deriving the velocity of a given position in a tissue using Optical Doppler Tomography was reported in detail by our group and we will provide a brief summary here^24^. Equation (1) is used to calculate the velocity difference in the axial direction:


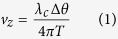


where *λ*_*c*_ = 1.31 *μm*, Δ*θ* = 2*π*, and T = *μ*s, giving the velocity difference *v*_*z*_ = 0.131 m/s. Hence in Figs 7 and 8, the true velocity can be calculated using *n* × 0.131 m/s, where *n* = 0 is the determined based on the peak and valley locations of the oscillation, and *n* in other regions then can be determined by their relative distance to *n* = 0. It must be understood, however, that the “velocity” herein is only related vector component in the direction of the laser light propagation, and the three components of the velocity vector cannot be calculated. Additionally, wrapped phase—causing the quasi-periodic patterns—allows the measurement of true axial velocity using the method described above. Video of Doppler B-frame images is shown at 10 fps in Supplementary Video 4. Although with lesser magnitude, the velocity distribution patterns can also be observed on the false vocal folds (Fig. 8).

## Discussion

Visualizing the vocal folds *in vivo* has evolved over three centuries beginning with sunlight and complex mirror systems to contemporary methods combining endoscopes with high-speed digital video hardware. Clinically, contemporary imaging technologies are limited because they only record images of the vocal fold surface. As the vocal folds vibrate, cross-sectional information about sub-epithelial processes would be invaluable to clinicians and scientists to who would use this information to better understand vocal fold pathology and vocal fold vibratory mechanics *in vivo*. Our group has worked extensively with VCSEL long-range OCT systems^30^,^31^,^32^,^33^. However, most investigations required contact or near-contact modes of evaluation under anesthesia, and hence vibration is not observed.

Imaging in the office and during phonation has been a long-term goal of several groups, including ours. There have been several reports of office-based laryngeal imaging OCT systems using either rigid transoral telescope or transnasal fiber-optic devices^10^,^17^,^18^,^19^,^20^,^21^,^22^. Fiber-optic approaches still require contact, however, and only image the free margin (superficial edge) of the fold. None of these devices have been able to image the cross sectional vocal fold structure *in vivo* during phonation. Previously, imaging the vibration of true vocal folds with proper spatial coherence has been demonstrated *ex vivo*^21^,^24^, and these studies clearly established the potential of OCT to capture high-resolution cross-sectional images of full true vocal fold vibration, Doppler shifts, and velocity distributions. While others—including ourselves—have previously imaged the vocal fold structure and vibration, here a clinically relevant, panoramic, cross-sectional view of laryngeal vibration *in vivo* is demonstrated for the first time.

*In vivo* transoral imaging of vibrating vocal folds has been accomplished, albeit with very poor image quality^11^,^17^,^18^,^19^,^20^,^21^,^22^. Lüerßen *et al*. demonstrated OCT vibratory patterns at an imaging speed of 10 fps^11^. We have previously demonstrated *in-vivo* phonating vocal folds using a 20 kHz swept source OCT system, imaging at 40 fps^22^. Donner *et al*. recently demonstrated a 16 kHz sweep rate OCT system to image both the static and phonating vocal folds at 25 fps^17^. None of these studies demonstrated images that provide clinically useful information on fold structure during phonation, and each scanned a relatively short lateral distance. In this study, we scan approximately 10 mm laterally on B-scan images. This allows for panoramas of the whole glottis including the false folds laterally. The issue of poor lateral resolution in previous studies was overcome here by using a higher A-line rate.

In most patients, the distance from any landmark within the oropharynx to the surface of the vocal folds is unknown, and the technician is tasked with estimating this distance and dynamically adjusting the reference path length of the OCT system on-the-fly in order to obtain images^22^. Due to the short imaging range of conventional OCT devices (approximately 1 mm), reliable imaging was a major challenge, and only limited success has been achieved in obtaining images of the vocal folds *in vivo* through a transoral technique^22^. The development of the long working distance in OCT imaging has been crucial to make significant progress in in-office OCT imaging, and we have accomplished this using a VCSEL source. With the present system, the imaging range is 12 mm, so estimating the distance from instrument tip to vocal fold within this range is straightforward and easy. Here the use of the long-range VCSEL source is transformative. It remedies the technical challenges that have limited the use of office-based laryngeal imaging in the previous iterations of this base technology^34^, and it does not require the sophisticated real-time signal processing and electromechanical drive systems of a feedback control system^17^.

One fundamental limitation with respect to OCT technology has been the depth of signal penetration. It has been previously noted that beneath the SLP layer, the signal intensity is greatly reduced as light enters and is backscattered by the ILP^4^,^12^. In our study, the DLP is visualized in some volunteers, but not all; this might be due to the variable thickness of the ILP^7^,^26^. Nevertheless, with our imaging system, we are confident that the epithelium and SLP can be observed under OCT, while at least a fraction of ILP can be seen. This is practically important as it demonstrates that OCT has the potential to evaluate an epithelial lesion to determine whether the basement membrane was breached (a hallmark of cancer), a finding that could previously only be determined using biopsy or OCT under general anesthesia. Other functional OCT imaging methods, such as polarization sensitive OCT, may permit further resolution of distinct tissue layers^34^,^35^.

Finally, technical challenges remain as with all transoral examinations. A low palatal arch, large base of tongue, and exaggerated gag reflex can limit the examination considerably. It is essential to have both a skilled examiner and a patient with optimal anatomy for all transoral approaches. It is also important for the examiner to keep in mind that the motion by the subjects’ larynx, neck, and head relative to the examiners’ hand and torso can influence the ability to pick up a stable OCT signal.

The concept of the Nyquist frequency must be considered in analyzing images of the phonating larynx. In the subject who was phonating at 850 Hz, as depicted in Fig. 4, the frame rate was 200 Hz and by being below the Nyquist frequency, we were unable image the vibratory pattern of the fold (Supplementary Video 1). For the subject who was phonating at 250 Hz as in Fig. 5, the lower phonating frequency allows the visualization of a more obvious mucosal wave. The lack of symmetry of the vocal folds occurs because the Nyquist frequency is 500 Hz, and our peak frame rate is 200 fps. It takes 5 ms to acquire one frame. However, the cord is vibrating at one cycle every 4 ms. The visualized mucosal wave is thus out of phase as one views the cords from left to right. Regardless, these images are able to show the vibratory pattern much more clearly (Supplementary Video 2). *In vivo* cross-sectional images of the glottic cycle at or near the patient’s fundamental frequency are visualized for the first time in an awake patient during phonation. While previously done with lower resolution B-mode ultrasound images by other groups^29^,^36^,^37^,^38^,^39^, we are able to observe the quasi-longitudinal wave moving along the vocal fold in the coronal plane during the glottis cycle with the OCT B-scan images.

Doppler images are able to be acquired by this system and equate to velocity distribution patterns of both false and true vocal folds^24^. This provides detailed information on the speed and direction of the propagating mucosal wave, which can be used to both validate computational models of vocal fold vibration and provide additional data on fold mechanical properties via elastography. Doppler image analysis is preferred over frequency analysis of pixel intensity because the sampled mucosal waves are complex due to the presence of multiple tissue layers—each with different mechanical properties within the vocal fold. Doppler imaging allows for real-time image analysis while still maintaining the ability to discriminate tissue layers without distortion. As we have demonstrated before, banding patterns in the Doppler signal are well visualized (Figs 7 and 8 and Supplementary Video 4)^24^. This allows the clinician to analyze the mucosal wave in another dimension: into the plane of the cross-sectional image. Current approaches, such as video stroboscopy and high-speed digital video, only provide information on surface feature changes. With infiltrating neoplastic processes such as laryngeal cancer, Doppler may be extremely valuable as it provides a means to visualize the mucosal wave into the depth of the focal fold, which would complement static structural images taken with OCT. For instance, if an early vocal fold cancer extends past the epithelial layer (the hallmark of invasive cancer), it would alter mucosal wave propagation, and a Doppler map would provide a stark means of image contrast based essentially upon both optical and mechanical contrast mechanisms.

With its point-of-care capability, OCT has numerous applications in enhancing the clinical management of vocal fold diseases and disorders of speech. It also provides a better understanding of the mechanics of vocal fold vibration. Training will be required for the operator to analyze and interpret the images, in much of the same way as with what has occurred with ultrasonography, now a widely adopted point-of-care imaging modality. Like ultrasound, real-time OCT imaging provides much more information for the clinician than static images, as regions of interest can be focally imaged and re-imaged during the examination. Furthermore, with the continued advances in hardware allowing higher image acquisition rates and processing power, 4D reconstruction of the vocal fold will be possible in a manner similar to what has been described in OCT imaging of embryonic hearts^40^. As with many other medical imaging modalities, 3D visualization is always more intuitive and easier for clinicians, and this will also allow for better understanding of the native vibratory behavior of the vocal cord under normal physiologic conditions. Recent innovations in 3D software packages could allow the physician to manipulate of these images in order to improve understanding of structural and functional images.

At this time, OCT in otolaryngology has not been widely embraced because of the cost and the lack of a paradigm shifting clinical application^41^. For true efficacy, OCT must be embraced as a point-of-care imaging method. Comparatively, ultrasound started initially as an expensive technology used only by highly trained individuals. As the ultrasound technology was more broadly adopted with the concomitant reduction in price per unit of the devices, training and expertise in terms of interpretation of imaging increased. It is likely that OCT will follow a similar adoption dynamic as the utility of this technology is further demonstrated.

## Conclusion

This is the first study to report images of the human vocal folds *in vivo* for both static cross-sectional images as well as functional data during phonation. OCT as an imaging modality offers high-resolution, high-speed, and panoramic images, and the analysis of these images holds great potential for evaluating epithelial lesions in many aspects of medicine. In terms of laryngology, OCT has the impact to revolutionize the standard of diagnosing and treating voice complaints. Currently, epithelial lesions of the vocal folds are diagnosed by biopsy in the operating room under general anesthesia. Combining cross-sectional and Doppler imaging has the potential to characterize lesions of the vocal folds by the depth of invasion in the office^42^. Further study will be focused on collecting data at a higher data acquisition rate to reduce aliasing effect and improve the data analysis of the OCT images. As a translational research topic, OCT has the potential to provide a more reliable and economical preoperative assessment when encountering lesions of the larynx.

## Additional Information

**How to cite this article**: Coughlan, C. *et al*. In vivo cross-sectional imaging of the phonating larynx using long-range Doppler optical coherence tomography. *Sci. Rep.* **6**, 22792; doi: 10.1038/srep22792 (2016).

## Supplementary Material

Supplementary Video 1

Supplementary Video 2

Supplementary Video 3

Supplementary Video 4

Supplementary Information
