# Supplementary material for: In vivo cross-sectional imaging of the phonating larynx using long-range Doppler optical coherence tomography
Source: Sci Rep. 2016 Mar 10;6:22792. doi: 10.1038/srep22792 (PMC4785353; doi:10.1038/srep22792)
Supplement: Supplementary Information [file srep22792-s5.pdf]

In vivo cross-sectional imaging of the phonating larynx using long-range Doppler optical coherence tomography

Carolyn A. Coughlan, MD[1], Li-dek Chou, MS[2,3], Joseph C. Jing, MS[2,4], Jason J. Chen, BS[2], Swathi Rangarajan, MS[3], Theodore H. Chang, BS[3], Giriraj K. Sharma, MD, MS[1], Kyoungrai Cho, MD[2,5], Donghoon Lee, MD[2,5], Julie A. Goddard, MD[1], Zhongping Chen, PhD[2,4*], Brian J.F. Wong, MD, PhD[1,2*]

[1] Department of Otolaryngology - Head and Neck Surgery, University of California, Irvine, School of Medicine
[2] Beckman Laser Institute & Medical Clinic, University of California, Irvine
[3] OCT Medical Imaging Inc.
[4] Department of Biomedical Engineering, University of California, Irvine
[5] Department of Otorhinolaryngology-Head & Neck Surgery, Sanggye Paik Hospital, Inje University, Korea
[*] Drs. Wong and Chen are the corresponding authors. Their emails are bjwong@uci.edu and z2chen@uci.edu respectively.

Supplementary Information

Supplementary Video 1. Video of B-frame images. The subject was phonating at approximately 850 Hz. The video is playing at 10 fps.

Supplementary Video 2. Video of B-frame images. The subject was phonating at approximately 250 Hz. The video is playing at 10 fps.

Supplementary Video 3. Real-time OCT with endoscopic video side by side. The left shows OCT images corresponding to the endoscopic video on the right.

Supplementary Video 4. Doppler B-scan video. The subject was phonating at about 250 Hz. The video is playing at 10 fps. The lateral dimension is shown in unadjusted aspect.
